# Multifaced risk factors and clinical impact of a deep Y descent in patients with heart failure irrespective of RV-PA coupling

**DOI:** 10.1016/j.ijcha.2024.101439

**Published:** 2024-06-10

**Authors:** Daisuke Harada, Takahisa Noto, Junya Takagawa

**Affiliations:** The Cardiology Division, Imizu Municipal Hospital, Toyama, Japan

**Keywords:** Heart failure, Right ventricular function, TAPSE, RV-PA coupling, Atrial fibrillation, Beta-blockers

## Abstract

**Background:**

A deep Y descent in the jugular venous pulse (JVP) is associated with diseases such as a decrease in right ventricular (RV) preload reserve. The present study investigated the relationship between RV-pulmonary arterial (PA) coupling and a deep Y descent, examined risk factors for a deep Y descent and clarified whether a deep Y descent was an independent risk factor for cardiac events irrespective of RV-PA coupling in patients with heart failure (HF).

**Methods:**

We enrolled 350 patients with HF who underwent echocardiography and JVP examination. A deep Y descent was identified by a deeper ‘Y’ descent than ‘X’ descent in the JVP waveform. We defined cardiac events of HF as follows: sudden death, death from HF, the emergent infusion of loop diuretics, or hospitalization for decompensated HF.

**Results and Conclusions:**

A deep Y descent and cardiac events were observed in 129 and 83 patients, respectively. The prevalence of a deep Y descent increased with decreases in the tricuspid annular plane systolic excursion (TAPSE)/systolic pulmonary arterial pressure (SPAP) ratio. Not only the TAPSE/SPAP ratio (odds ratio,0.756 per0.1 mm/mmHg, 95 %confidence interval [CI], 0.660–0.866, p < 0.001), but also age, atrial fibrillation, and the use of beta-blockers were independent factors for a deep Y descent in multivariate logistic model. Multivariate Cox hazard model demonstrated that a deep Y descent was for cardiac events in patients with HF (Hazard ratio,2.682, 95 %CI, 1.599–4.497, p < 0.001) irrespective of the TAPSE/SPAP ratio. The development of therapeutic strategies based on central venous waveform may be needed for patients with HF.

## 1 Introduction

Heart failure (HF) is a rapidly growing public health issue worldwide due to increases in life expectancy [1], [2]. A more detailed understanding of the pathophysiology of HF has resulted in many researchers recognizing the importance of right ventricular (RV) function in patients with HF [3], [4]. Cardiac catheterization has traditionally been used to examine RV function. The process of RV adaptative responses to pulmonary hypertension is now attracting interest, particularly in patients with pulmonary arterial (PA) hypertension [3]. If pulmonary hypertension persists, the RV preload reserve gradually decreases and eventually becomes limited, which is an unfavorable state in RV pressure loading. Since pulmonary hypertension due to left-sided HF also results in pressure on right ventricle, similar adaptations occur in right ventricle as those in PA hypertension. Therefore, RV-PA coupling and the RV preload reserve need to be assessed in patients with HF. However, performing a catheter examination on older patients with HF may be challenging due to its invasive nature. As an alternative, echocardiography is an excellent tool for measuring cardiac function and the tricuspid annular plane systolic excursion (TAPSE)/systolic pulmonary arterial pressure (SPAP) ratio, an indicator of RV-PA coupling, has been developed [5]. If there are issues with cardiac function assessment via echocardiography, indices of the RV preload reserve have not yet been established [6], and, thus, the relationship between RV-PA coupling and the RV preload reserve in patients with HF remains unclear. The jugular venous pulse (JVP) is non-invasive, easily examined in outpatient clinics, and reflects the characteristics of right ventricle during the diastolic phase [7], [8]. We previously demonstrated that the presence of a deeper Y descent than the X descent in the JVP waveform suggested a decreased RV preload reserve, which has a negative impact on the clinical outcomes of HF [9], [10]. The JVP waveform will provide insights into the relationship between RV-PA coupling and the RV preload reserve in patients with HF. Moreover, the identification of risk factors for a decrease in the RV preload reserve will be significant. Therefore, the present study examined the relationship between RV-PA coupling and a deep Y descent and attempted to identify patient’s characteristics associated with a deep Y decent in patients with HF. We also investigated whether a deep Y descent was an independent risk factor for cardiac events irrespective of RV-PA coupling.

## 2 Methods

### 2.1 Study design

**Fig. 1:**
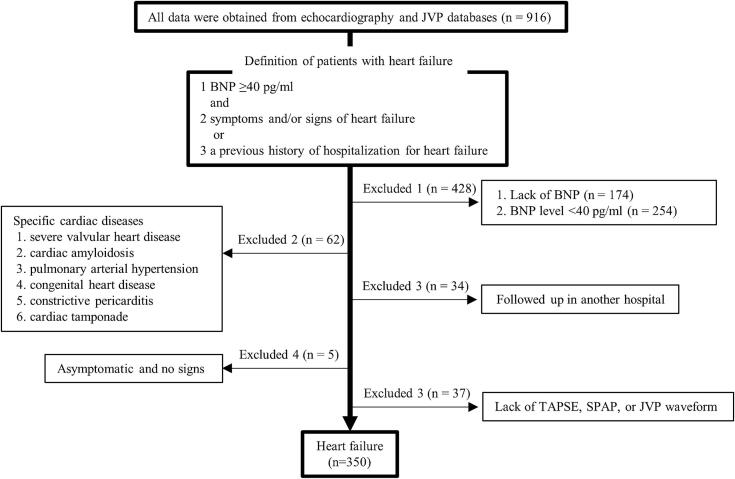
Study flow chart. BNP, brain natriuretic peptide; JVP, jugular venous pulse; TAPSE, tricuspid annular plane systolic excursion; SPAP, systolic pulmonary arterial pressure.

The institutional human subject review committee of our institute approved the present study. All data were retrospectively obtained from echocardiographic and JVP databases and medical records between April 2017 and March 2021. A study flow chart is shown in Fig. 1. All patients enrolled in the present study were Japanese. We defined patients with HF as those with a brain natriuretic peptide (BNP) level ≥ 40 pg/ml and symptoms and/or signs of or prior hospitalization for HF [11]. Patients were excluded if they lacked data on BNP or its level was < 40 pg/ml. We excluded 62 patients clinically diagnosed with specific cardiac diseases, such as severe valvular heart disease, cardiac amyloidosis, PA hypertension, congenital heart disease, constrictive pericarditis, and cardiac tamponade, which have a poor prognosis and/or are treated by invasive procedures, such as surgery. Patients were excluded if they were unable to be followed up in our hospital and had no symptoms or signs of HF. Following the exclusion of patients for whom TAPSE, SPAP, or the JVP waveform were not measured, 350 patients were ultimately enrolled in the present study. We classified HF into three categories according to the left ventricular ejection fraction (LVEF) [HF with reduced EF (HFrEF), HF with mid-range EF (HFmrEF), and HF with preserved EF (HFpEF)] and comparisons were performed between patients with HFmr/rEF (HF with mid-range or reduced EF) and with HFpEF. A multivariate analysis was also conducted to identify independent variables for a deep Y descent and cardiac events. Based on the arrangement of our hospital, all patients provided their informed consent. The present study complied with the Declaration of Helsinki.

### 2.2 Evaluation of echocardiography

Echocardiography was assessed as described in our previous study [10]. An echocardiographic examination (Vivid 7, General Electric Healthcare, Wauwatosa, WI, USA) was performed with reference to the guidelines [12], [13], [14]. Left ventricular (LV) end-diastolic and end-systolic volumes were measured using a modification of Simpson’s method. LVEF was calculated as the stroke volume divided by the end-diastolic volume. To evaluate diastolic properties, we measured early diastolic velocities (e’) using pulsed-wave tissue Doppler from the apical view. We also measured septal and lateral E/e’ and averaged the values obtained for a more reliable assessment of LV diastolic function and filling pressure [12]. The left atrial volume index was obtained using the biplane method from both the apical 4- and 2-chamber views [13]. The LV mass was calculated using the Devereux formula and was divided by surface area [13]. TAPSE was measured from the apical four-chamber view. The tricuspid regurgitant jet was detected using the continuous Doppler technique to measure RV systolic pressure [14]. We regarded RV systolic pressure as SPAP because of the absence of a gradient across the pulmonic valve and RV outflow tract. The severity of valvular heart disease was examined according to the guidelines [15]. In patients with atrial fibrillation, velocity measurements were estimated from 10 cardiac cycles [12].

### 2.3 JVP

#### 2.3.1 Method to measure JVP

JVP is a non-invasive tool that detects the waveform of the internal jugular vein reflecting the waveform of the right atrium. The methods employed herein to measure and judge JVP were similar to those in our previous study [7]. JVP was recorded in the supine position by well-trained cardiac sonographers. A pulse-wave transducer (TY-306, Fukuda Denshi, Tokyo, Japan) was placed over the neck, above and to the right of the junction of the right clavicle and the manubrium sterni, and held in place manually. To exclude respiratory variations, subjects held their breath during expiration while JVP was measured.

#### 2.3.2 The JVP waveform and its assessment method

**Fig. 2:**
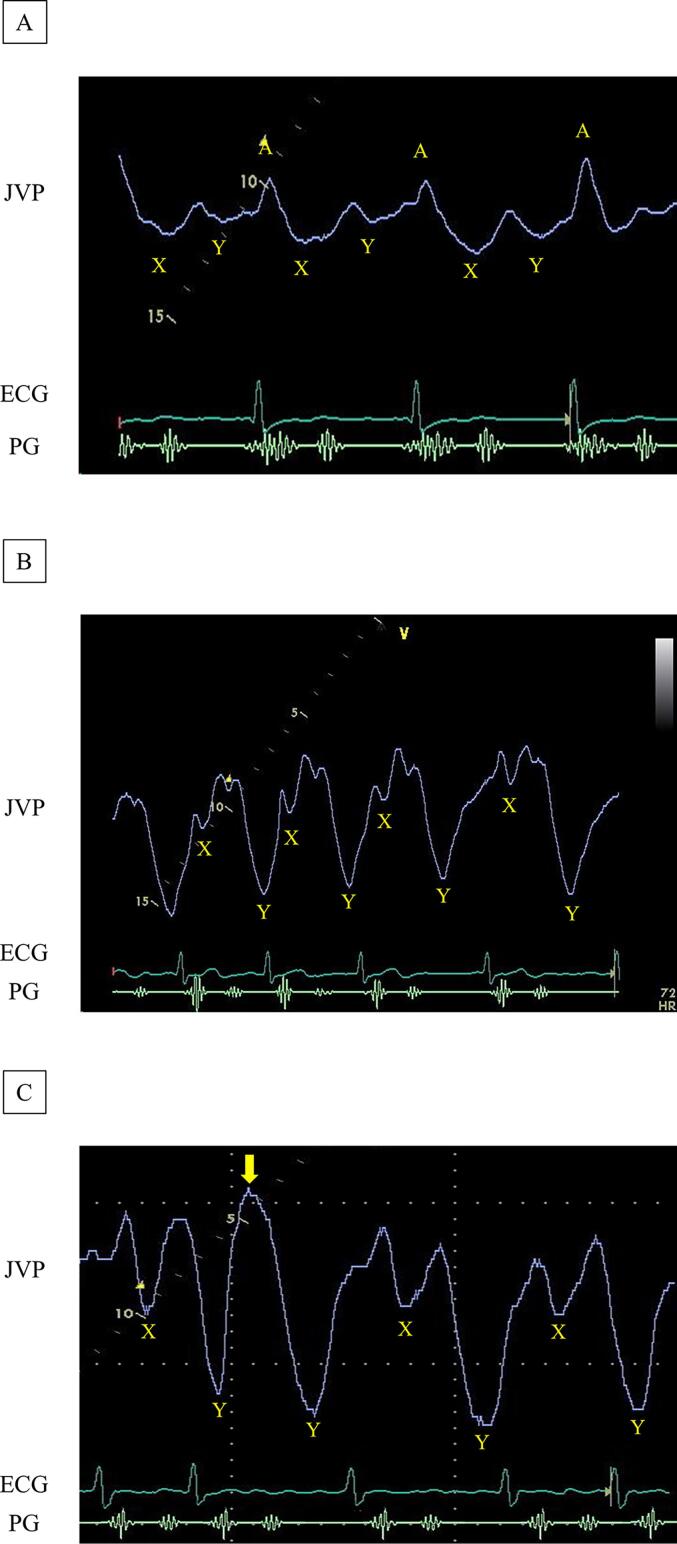
Jugular venous pulse. (A) Normal Y descent. (B) and (C) Deep Y descent. A 68-year-old man had a normal jugular venous pulse waveform characterized by the highest A wave and lowest X descent within one cardiac cycle (A). A 87-year-old woman had atrial fibrillation and moderate tricuspid regurgitation. A deeper Y descent than the X descent was observed in the jugular venous pulse (B). A 78-year-old woman had atrial fibrillation and mild tricuspid regurgitation. The preceding R-R interval was short and the X descent disappeared in the second heartbeat (yellow arrow). A deep Y descent was confirmed in other cardiac cycles (C). ECG, electrocardiogram; JVP, jugular venous pulse; PG, phonocardiography.

JVP are shown in Fig. 2. X and Y descents were distinguished based on whether they were located before or after the second sound on a phonocardiogram. A normal jugular venous waveform, characterized by the highest ‘A’ wave and lowest ‘X’ descent within one cardiac cycle, is shown in Fig. 2A. In contrast, the JVP waveform, characterized by a dominant ‘Y’ descent with a lower nadir than that of the ‘X’ descent, is shown in Fig. 2B and was judged as an abnormal waveform based on the findings of a previous study [16]. Therefore, cardiologists compared the relative depth of the nadirs of the ‘X’ and ‘Y’ descent and judged that JVP represented an abnormal pattern where the nadir of the ‘Y’ descent was deeper than that of the ‘X’ descent in the present study. Although atrial fibrillation *per se* causes the shortening of and a reduction in the depth of the X descent, atrial fibrillation alone does not cause the disappearance of the X descent and the degree of which is somewhat variable from patient to patient [16]. However, the X descent disappears in some cases, particularly with tricuspid regurgitation or when the preceding R-R interval becomes shortened (Fig. 2 C). Therefore, cases of atrial fibrillation were recorded for at least 5 cardiac cycles, and the depths of the X and Y descents were compared. Additional waveforms of JVP are shown in supplementary file 1.

### 2.4 Documentation of endpoints

All 350 patients were followed up at our hospital. We defined cardiac events as follows: sudden death, death from HF, the emergent infusion of loop diuretics to treat lung congestion, or hospitalization for the deterioration of HF. These cardiac events were reported and adjudicated by cardiovascular specialists at our hospital.

### 2.5 Statistical analysis

Numerical data were expressed as a mean (standard deviation) or median (interquartile range). The Student’s *t* test, Mann-Whitney *U* test, and Fisher’s exact test were used to compare numerical data and non-parametric data between the groups. A logistic model was created to identify independent factors for a deep Y descent. In outcome analyses, we used Cox proportional hazard models to examine independent relationships between features and cardiac events. In multivariate logistic and Cox proportional hazard models, we selected variables with a P value < 0.2 in univariate analyses. The multicollinearity of variables for a deep Y descent was assessed. The correlation matrix between selected variables for cardiac events was also estimated (Supplementary file 2 and 3) [17], [18]. Due to the large number of explanatory variables relative to the sample size, we constructed stepwise backward models based on p-values. Significance was established at p < 0.05. Statistical analyses were performed using EZR (Saitama Medical Center, Jichi Medical University, Saitama, Japan) [19].

## 3 Results

### 3.1 Patient characteristics

Patient characteristics are shown in Table 1. Body mass index, mean blood pressure, and the ratio of hypertension were lower in patients with HFmr/rEF than in those with HFpEF. The ratio of males, pleural effusion, old myocardial infarction, prior hospitalization for decompensated HF, and medications for HF, and BNP levels.

**Table 1:** Patient characteristics according to the heart failure phenotype.

	Total n = 350	HFmr/rEF n = 67	HFpEF n = 283	p-value
Age, years	78 (70–84)	75 (68–82)	78 (71–83)	0.071
Male, %	184 (53)	47 (70)	137 (48)	0.002
Body mass index, kg/m^2^	23 (21–25)	22 (21–25)	23 (21–26)	0.029
Heart rate, bpm	65 (58–76)	66 (58–83)	65 (58–75)	0.301
Mean blood pressure, mmHg	90 ± 14	86 ± 14	91 ± 14	0.004
HF phenotype at the entry point				
HFrEF	34 (10)	34 (51)	0 (0)	<0.001
HFmrEF	33 (9)	33 (49)	0 (0)	
HFpEF	283 (81)	0 (0)	283 (1 0 0)	
HF symptoms and signs				
Dyspnea	332 (95)	66 (99)	266 (94)	0.216
Leg edema	181 (52)	41 (61)	140 (49)	0.103
Pleural effusion	113 (32)	35 (52)	78 (28)	<0.001
Hypertension, %	284 (81)	46 (69)	238 (84)	0.005
Diabetes mellitus, %	84 (24)	14 (21)	70 (25)	0.633
Old myocardial infarction, %	56 (16)	18 (27)	38 (13)	0.010
Prior hospitalization for DHF, %	143 (41)	43 (64)	100 (35)	<0.001
Atrial fibrillation, %	105 (30)	15 (22)	90 (32)	0.141
ACEI/ARB, %	257 (73)	57 (85)	200 (71)	0.020
MRA, %	111 (32)	34 (51)	77 (27)	<0.001
ARNI, %	7 (2)	5 (7)	2 (1)	0.003
Beta blockers, %	237 (68)	63 (94)	174 (61)	<0.001
Ca antagonist, %	141 (40)	14 (21)	127 (45)	0.572
Loop diuretics, %	160 (46)	50 (75)	110 (39)	<0.001
SGLT-2 inhibitor, %	33 (9)	8 (12)	25 (9)	0.485
Estimated GFR, ml/min	59 (47–73) (n = 342)	58 (41–70) (n = 66)	60 (47–73) (n = 276)	0.283
Hemoglobin level, g/dl	13 (11–14) (n = 343)	13 (12–15) (n = 66)	13 (11–14) (n = 277)	0.141
Brain natriuretic peptide, pg/ml	153 (79–326)	395 (145–624)	133 (75–272)	<0.001
Cardiac events, %	83 (24)	31 (46)	52 (18)	<0.001

Data are shown as the number of patients (%), average ± standard deviation, or a median (interquartile range). ACEI/ARB, angiotensin-converting enzyme inhibitors/angiotensin receptor blockers; ARNI, angiotensin receptor-neprilysin inhibitor; DHF, decompensated heart failure; GFR, glomerular filtration rate; HF, heart failure; HFmrEF, heart failure with mid-range ejection fraction; HFmr/rEF, heart failure with mid-range or reduced ejection fraction; HFpEF, heart failure with preserved ejection fraction; HFrEF, heart failure with reduced ejection fraction; MRA, mineralocorticoid receptor antagonist; SGLT-2, sodium glucose co-transporter 2.

were higher in patients with HFmr/rEF than in those with HFpEF. During a mean follow-up period of 35 ± 19 months, 83 patients had cardiac events of HF. The development of cardiac events was also higher in patients with HFmr/rEF than in those with HFpEF. Out of 350 patients, 153 had undergone echocardiography at our hospital prior to the entry point, allowing for the longitudinal observation of LVEF changes. There were 36 patients classified as HF with recovered EF, 107 classified as HF with unchanged EF, and 10 classified as HF with worsened EF at the entry point (Supplementary file 4).

### 3.2 Cardiac function and morphology

Cardiac function and morphology are shown in Table 2. LVEF and TAPSE/SPAP were lower, mean mitral e’ was slower and TAPSE was shorter in patients with HFmr/rEF than in those with HFpEF. The LV mass index and LV end diastolic dimension were larger and the mean mitral E/e’ ratio and the ratio of moderate mitral regurgitation were higher in patients with HFmr/rEF than in those with HFpEF. Patients with HFpEF and HFmr/rEF had similar incidence of a deep Y descent.

**Table 2:** Cardiac morphology and function according to the heart failure phenotype.

	Total n = 350	HFmr/rEF n = 67	HFpEF n = 283	p-value
Left heart				
LAVI, ml/m^2^	46 (35–62)	47 (37–73)	46 (35–60)	0.322
LVMI, g/m^2^	118 (97–144)	140 (119–180)	114 (95–138)	<0.001
LVEF, %	64 (54–72)	40 (33–45)	67 (61–73)	<0.001
LVEDD, mm	47 (43–52)	55 (50–61)	46 (43–50)	<0.001
Mean mitral e’, cm/s	6.0 (4.8–7.9) (n = 349)	4.8 (3.8–5.7) (n = 66)	6.5 (5.1–8.3) (n = 283)	<0.001
Mean mitral E/e’ ratio	12 (10–17) (n = 349)	16 (12–21) (n = 66)	11 (9–15) (n = 283)	<0.001
Moderate MR, %	55 (16)	19 (28)	36 (13)	0.003
Right heart				
RVOT, mm	29 (27–32)	30 (26–32)	29 (27–32)	0.586
TAPSE, mm	19 (17–22)	18 (15–20)	20 (17–23)	0.001
SPAP, mmHg	25 (21–30)	24 (22–38)	25 (21–29)	0.306
TAPSE/SPAP ratio	0.76 (0.60–0.95)	0.67 (0.48–0.85)	0.78 (0.63–0.99)	<0.001
Moderate TR, %	36 (10)	4 (6)	32 (11)	0.264
Deep Y descent, %	129 (37)	24 (36)	105 (37)	0.889
IVC, mm	13 (10–17) (n = 344)	14 (10–17) (n = 67)	13 (10–16) (n = 277)	0.437

Data are shown as the number of patients, (%) or a median (interquartile range). HFmr/rEF, heart failure with mid-range or reduced ejection fraction; HFpEF, heart failure with preserved ejection fraction; IVC, inferior vena cava; LAVI, left atrial volume index; LVEDD, left ventricular end diastolic dimension; LVEF, left ventricular ejection fraction; LVMI, left ventricular mass index; MR, mitral regurgitation; RV, right ventricle; RVOT, right ventricular outflow tract; SPAP, systolic pulmonary arterial pressure; TAPSE, tricuspid annular plane systolic excursion; TR, tricuspid regurgitation.

### 3.3 Prevalence of a deep Y descent according to the TAPSE/SPAP ratio

**Fig. 3:**
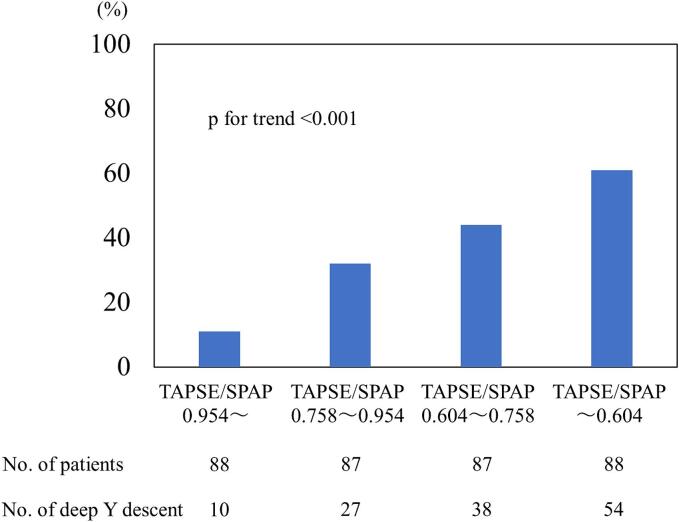
The frequency of a less-distensible right ventricle based on quartiles of the TAPSE/SPAP ratio. SPAP, systolic pulmonary arterial pressure; TAPSE, tricuspid annular plane systolic excursion.

The prevalence of a deep Y descent according to the TAPSE/SPAP ratio is shown in Fig. 3. The prevalence of a deep Y descent increased and was accompanied by a decrease in the TAPSE/SPAP ratio; however, a deep Y descent was observed from the stage at which the TAPSE/SPAP ratio was preserved.

### 3.4 Univariate and multivariate analyses of a deep Y descent

The results of univariate and multivariate analyses of a deep Y descent are shown in Table 3. Multivariate logistic models identified age (odds ratio [OR], 1.060 per 1 year, 95 % confidence interval [CI], 1.020–1.090, p = 0.004), atrial fibrillation (OR, 26.00, 95 % CI, 12.60–53.50, p < 0.001), the use of beta-blockers (OR, 2.160, 95 % CI, 1.080–4.310, p = 0.030), and the TAPSE/SPAP ratio (OR, 0.756 per 0.1 mm/mmHg, 95 % CI, 0.660–0.866, p < 0.001) as independent factors for a deep Y descent.

**Table 3:** Results of univariate and multivariate analyses of a deep Y descent.

	Univariate analysis		Multivariate analysis stepwise reduction	
	Odds ratio (Confidence interval)	p-value	Odds ratio (Confidence interval)	p-value
Age, per 1 year	1.020 (0.998–1.050)	0.070	1.060 (1.020–1.090)	0.004
Male	1.170 (0.757–1.810)	0.480		
Body mass index, per 1 kg/m^2^	1.040 (0.977–1.100)	0.230		
Heart rate, per 1 bpm	1.020 (1.000–1.030)	0.020		
Mean blood pressure, per 1 mmHg	1.010 (0.991–1.020)	0.424		
Hypertension	0.642 (0.373–1.100)	0.110		
Diabetes mellitus	0.762 (0.453–1.280)	0.305		
Old myocardial infarction	0.943 (0.520–1.710)	0.847		
Prior hospitalization for DHF	1.450 (0.931–2.250)	0.101		
Atrial fibrillation	23.40 (12.70–42.80)	<0.001	26.00 (12.60–53.50)	<0.001
ACEI/ARB	0.702 (0.432–1.140)	0.152		
MRA	1.850 (1.170–2.940)	0.009		
ARNI	2.330 (0.512–10.60)	0.274		
Beta blockers	1.860 (1.150–3.040)	0.012	2.160 (1.080–4.310)	0.030
Ca antagonist	0.859 (0.550–1.340)	0.503		
Loop diuretics	2.340 (1.500–3.640)	<0.001		
SGLT-2 inhibitor	1.480 (0.720–3.060)	0.284		
Estimated GFR, per 1 ml/min	0.995 (0.985–1.010)	0.333		
Hemoglobin level, per 1 g/dl	1.040 (0.932–1.150)	0.502		
Brain natriuretic peptide, per 1 pg/ml	1.000 (1.000–1.000)	0.016		
LAVI, per 1 ml/m^2^	1.040 (1.030–1.050)	<0.001		
LVMI, per 1 g/m^2^	1.000 (0.997–1.010)	0.456		
LVEF, per 1 %	0.996 (0.981–1.010)	0.663		
LVEDD, per 1 mm	0.996 (0.965–1.030)	0.786		
Mean mitral e’, per 1 cm/s	1.260 (1.130–1.390)	<0.001		
Mean mitral E/e’ ratio, per 1	1.040 (1.010–1.080)	0.006		
Moderate MR	1.990 (1.110–3.560)	0.020		
RVOT, per 1 mm	1.040 (0.994–1.100)	0.085		
TAPSE/SPAP ratio, per 0.1 mm/mmHg	0.688 (0.617–0.766)	<0.001	0.756 (0.660–0.866)	<0.001
Moderate TR	6.240 (2.83–13.70)	<0.001		
IVC, per 1 mm	1.190 (1.120–1.260)	<0.001		

ACEI/ARB, angiotensin-converting enzyme inhibitors/angiotensin receptor blockers; ARNI, angiotensin receptor-neprilysin inhibitor; DHF, decompensated heart failure; GFR, glomerular filtration rate; IVC, inferior vena cava; LAVI, left atrial volume index; LVEDD, left ventricular end diastolic dimension; LVEF, left ventricular ejection fraction; LVMI, left ventricular mass index; MR, mitral regurgitation; MRA, mineralocorticoid receptor antagonist; RVOT, right ventricular outflow tract; SGLT-2, sodium glucose co-transporter 2; SPAP, systolic pulmonary arterial pressure; TAPSE, tricuspid annular plane systolic excursion; TR, tricuspid regurgitation.

### 3.5 Univariate and multivariate analyses of cardiac events

The results of univariate and multivariate analyses of cardiac events are shown in Table 4. Multivariate Cox hazard models demonstrated that prior hospitalization for decompensated HF (OR, 1.943, 95 % CI, 1.202–3.140, p = 0.007), the use of loop diuretics (OR, 3.565, 95 % CI, 1.953–6.508, p < 0.001), hemoglobin levels (OR, 0.825 per 1 g/dl, 95 % CI, 0.749–0.909, p < 0.001), BNP (OR, 1.001 per 1 pg/ml, 95 % CI, 1.000–1.001, p < 0.001), TAPSE/SPAP ratio (OR, 0.816 per 0.1 mm/mmHg, 95 % CI, 0.729–0.912, p < 0.001), and deep Y descent (OR, 2.682, 95 %CI, 1.599–4.497, p < 0.001) were independent factors for cardiac events with HF.

**Table 4:** Results of univariate and multivariate analyses of cardiac events.

	Univariate analysis		Multivariate analysis stepwise reduction	
	Hazard ratio (Confidence interval)	p-value	Hazard ratio (Confidence interval)	p-value
Age, per 1 year	1.093 (1.063–1.124)	<0.001		
Male	1.085 (0.705–1.671)	0.711		
Body mass index, per 1 kg/m^2^	0.933 (0.873–0.998)	0.045		
Heart rate, per 1 bpm	1.020 (1.006–1.034)	0.004		
Mean blood pressure, per 1 mmHg	0.987 (0.971–1.003)	0.109		
Hypertension	0.610 (0.372–1.002)	0.051		
Diabetes mellitus	0.757 (0.444–1.290)	0.306		
Old myocardial infarction	0.993 (0.549–1.796)	0.982		
Prior hospitalization for DHF	2.844 (1.822–4.439)	<0.001	1.943 (1.202–3.140)	0.007
Atrial fibrillation	1.518 (0.977–2.359)	0.063		
ACEI/ARB	1.090 (0.665–1.789)	0.732		
MRA	2.568 (1.669–3.951)	<0.001		
ARNI	1.857 (0.455–7.576)	0.388		
Beta blockers	1.047 (0.655–1.674)	0.848		
Ca antagonist	0.788 (0.504–1.234)	0.298		
Loop diuretics	7.022 (4.061–12.14)	<0.001	3.565 (1.953–6.508)	<0.001
SGLT-2 inhibitor	0.808 (0.372–1.752)	0.589		
Estimated GFR, per 1 ml/min	0.974 (0.963–0.986)	<0.001		
Hemoglobin level, per 1 g/dl	0.782 (0.713–0.858)	<0.001	0.825 (0.749–0.909)	<0.001
Brain natriuretic peptide, per 1 pg/ml	1.001 (1.001–1.001)	<0.001	1.001 (1.000–1.001)	<0.001
LAVI, per 1 ml/m^2^	1.015 (1.008–1.022)	<0.001		
LVMI, per 1 g/m^2^	1.007 (1.002–1.012)	0.003		
LVEF, per 1 %	0.965 (0.951–0.978)	<0.001		
LVEDD, per 1 mm	1.022 (0.991–1.053)	0.173		
Mean mitral e’, per 1 cm/s	0.856(0.770–0.951)	0.004		
Mean mitral E/e’ ratio, per 1	1.054 (1.032–1.077)	<0.001		
Moderate MR	1.072 (0.593–1.938)	0.818		
RVOT, per 1 mm	1.031 (1.013–1.050)	<0.001		
TAPSE/SPAP ratio, per 0.1 mm/mmHg	0.663 (0.596–0.738)	<0.001	0.816 (0.729–0.912)	<0.001
Moderate TR	3.843 (2.359–6.261)	<0.001		
Deep Y descent	3.691 (2.357–5.780)	<0.001	2.682 (1.599–4.497)	<0.001
IVC, per 1 mm	1.132 (1.079–1.189)	<0.001		

ACEI/ARB, angiotensin-converting enzyme inhibitors/angiotensin receptor blockers; ARNI, angiotensin receptor-neprilysin inhibitor; DHF, decompensated heart failure; GFR, glomerular filtration rate; IVC, inferior vena cava; LAVI, left atrial volume index; LVEDD, left ventricular end diastolic dimension; LVEF, left ventricular ejection fraction; LVMI, left ventricular mass index; MR, mitral regurgitation; MRA, mineralocorticoid receptor antagonist; RVOT, right ventricular outflow tract; SGLT-2, sodium glucose co-transporter 2; SPAP, systolic pulmonary arterial pressure; TAPSE, tricuspid annular plane systolic excursion; TR, tricuspid regurgitation.

## 4 Discussion

In the present study, the main results demonstrated using JVP waveform were as follows. As the TPASE/SPAP ratio decreased, the prevalence of a deep Y descent increased. Not only the TAPSE/SPAP ratio, but also other factors were associated with a deep Y descent. A deep Y descent was associated with cardiac events in patients with HF irrespective of the TAPSE/SPAP ratio.

### 4.1 The clinical significance of a deeper Y descent than the X descent in the JVP waveform

The Y descent is rapid and deep under conditions associated with rapid RV filling in early diastole. A Y descent deeper than the X descent is classically observed in representative diseases such as tricuspid regurgitation, constrictive pericarditis and restrictive cardiomyopathy [16], [20]. In patients with these specific cardiac diseases, the RV preload reserve is smaller than that in normal subjects because of volume overload or a stiffer pericardium and right ventricle. Moreover, we previously reported that the relative depth of the nadirs of X and Y descents in the waveforms between right atrial pressure and JVP were similar, while the stroke volume was lower in patients with a deep Y descent than in those with a normal Y descent [7], [8]. Furthermore, a negative relationship between the stroke volume index and pulmonary vascular resistance index was observed in the deep Y descent group, but not in the normal Y descent group [8]. If the RV preload is recruitable, stroke volume does not decrease in response to an increase in the RV afterload, namely, a preload-afterload mismatch in right ventricle cannot occur. The RV preload reserve was smaller in patients with a deep Y descent than in those with a normal Y descent. Therefore, a deep Y descent in the JVP waveform serves as an index for a decreased RV preload reserve.

### 4.2 The relationship between the TAPSE/SPAP ratio and a deep Y descent

RV concentric hypertrophy is a compensatory mechanism against continuous increases in the RV afterload. However, when this compensatory mechanism fails and RV contractility no longer increases to match the RV afterload, RV eccentric hypertrophy occurs, the RV preload reserve is gradually decreased and, ultimately, becomes limited [3]. The present study showed that the prevalence of a deep Y descent increased with decreases in the TAPSE/SPAP ratio and RV maladaptation occurred in HF characterized by secondary pulmonary hypertension. To the best of our knowledge, this is the first study to non-invasively investigate the relationship between RV-PA coupling and the RV preload reserve using echocardiographic indices and the JVP waveform. However, the decrease observed in the TAPSE/SPAP ratio alone cannot explain all aspects of the deep Y descent.

### 4.3 Factors associated with a deep Y descent

The present study demonstrated that several factors were associated with a deep Y descent. Age-related changes such as increased RV afterload, cell loss and fibrosis in the right ventricle have been reported [21], [22]. These changes indirectly or directly affect RV diastolic function and the preload reserve and are fundamentally irreversible and intractable. Few studies have examined the effectiveness of beta-blockers for the RV preload reserve in elderly patients with HF. An invasive examination of the acute effects of an infusion with the beta-blocker, esmolol in patients with PA hypertension showed increases in the RV end diastolic volume instead of heart rate with exercise [23]. Moreover, Ferlinz et al. reported that the long-term use of the beta-blockers, oxprenolol and propranolol, increased RV end-diastolic pressure and decreased RVEF in patients with essential hypertension, which indicated that the long-term use of beta-blockers impairs RV diastolic function [24]. Therefore, the negative chronotropic and inotropic effects of beta-blockers may lead to a decreased RV preload reserve and a deep Y descent due to RV volume overload. Atrial fibrillation affects hemodynamics in patients with HF. The RV preload becomes predominantly dependent on the rapid filling phase indicated by the Y descent and compensatory mechanisms, such as increasing the RV preload and/or heart rate, may occur to overcome decreases in the stroke volume derived from the elimination of atrial contractions. As described above, chronic increases in the RV volume leads to RV eccentric hypertrophy. Heart rate affects the ventricular filling time in diastole. Tachycardia shortens the ventricular filling time and induces incomplete ventricular relaxation, which may contribute to an upward shift in the RV end diastolic pressure–volume relationship. On the other hand, bradycardia prolongs the ventricular filling time and induces an increase in the ventricular end diastolic volume. These actions may contribute to a rightward shift in the RV end diastolic pressure–volume relationship. Although a previous study focused on left ventricle, atrial fibrillation is also related to ventricular myocardial fibrosis [25]. Therefore, atrial fibrillation is associated with deep Y descent.

### 4.4 Clinical implications

It currently remains unclear whether decreased RV preload reserve is associated with clinical outcomes in patients with HF during RV adaptation or maladaptation to pulmonary hypertension independent of RV contractility [3]. To the best of our knowledge, this is the first study to demonstrate that a deep Y descent was a risk factor for clinical outcomes in patients with HF irrespective of the TAPSE/SPAP ratio. This study also showed that the phenotype of HF did not affect the prevalence of a deep Y descent. Although guidelines have different treatment strategies for HF based on LVEF [11], it may be necessary to consider those that also take into account RV preload reserve. Therefore, further studies are needed to not only evaluate RV-PA coupling, but also to assess RV preload reserve and investigate the features contributing to these impairments for the management of patients with HF. Pulmonary hypertension may be a target for treatment in patients with HF; however, the effectiveness of PA vasoactive drugs remains controversial [26]. Phosphodiesterase-3 inhibitors exert the acute effects of decreasing pulmonary vascular resistance and increasing the stroke volume in patients with HF [27]. However, the effectiveness of phosphodiesterase-3 inhibitors for improving clinical outcomes in patients with HFrEF has yet to be confirmed [26], [28]. The use of inotropic agents in patients with HFpEF who develop decompensated right HF and a decrease in the TAPSE/SPAP ratio may need to be reconsidered. Atrial fibrillation is one of the targets for the treatment of HF because the clinical outcomes of patients with a maintained sinus rhythm are better than those of patients with atrial fibrillation [29]. A previous study also reported that the JVP waveform varied depending on atrial fibrillation or a sinus rhythm [30]. Therefore, a deep Y descent due to atrial fibrillation is reversible and the maintenance of a sinus rhythm contributes to better clinical outcomes in patients with HF by improving the RV preload reserve. The presence of a deep Y descent needs to be suspected in cases in which the worsening of right HF occurs with the administration of beta-blockers because cardiac output becomes more dependent on heart rate in patients with a deep Y descent [8]. Beta-blockers have no beneficial effect in patients with HFpEF and a deep Y descent [31]. Therefore, careful consideration of the use of beta-blockers is needed for patients in whom their administration may exacerbate right HF.

### 4.5 Study limitation

The present study has several limitations. Some patients, such as obese patients with limited windows or fatty neck, may have been underrepresented due to a lack of satisfactory imaging of echocardiography and JVP. Patients with tachycardia may also have been excluded because of difficulty in separating the X descent and Y descent of JVP. The lack of data on BNP levels or RV indices of echocardiography and the JVP waveform could contribute to a selection bias. The small number of patients with a deep Y descent and cardiac events may have limited the number of explanatory variables to create multivariate analysis models. Furthermore, overfitting might have occurred because the multivariate analysis models were created using a stepwise backward elimination method based on p-values. Thus, model validation may be needed. We previously reported that the RV preload reserve was smaller in patients with a deep Y descent than in those with a normal Y descent; however, a deep Y descent is merely a qualitative indicator of decreased RV preload reserve, and the extent of the decrease in the RV preload reserve cannot be assessed. The use of the overall contractility of the right ventricle, such as change in the RV fractional area, rather than just longitudinal contractility, such as TAPSE, reflects RV-PA coupling more accurately. Angiotensin receptor-neprilysin and sodium glucose co-transporter 2 inhibitors, which improve the clinical outcomes of patients with HF [32], were rarely prescribed to patients with HF in our cohort because they were only approved for the treatment of HF in Japan from August 2020 and November 2020, respectively. Since this was a cross-sectional and retrospective study, causality was not fully identified. Therefore, further clinical prospective studies are warranted to elucidate the impact of a deep Y descent in patients with HF.

The prevalence of a deep Y descent increased with impairments in RV-PA coupling. A deep Y descent was a risk factor for cardiac events in patients with HF irrespective of RV-PA coupling. To further improve the prognosis of patients with HF, the development of therapeutic strategies based on the central venous waveform may be needed in patients with HF in the future.

### CRediT authorship contribution statement

**Daisuke Harada:** Writing – review & editing, Writing – original draft, Validation, Formal analysis, Data curation, Conceptualization. **Takahisa Noto:** Data curation. **Junya Takagawa:** Validation, Data curation.

## Declaration of competing interest

The authors declare that they have no known competing financial interests or personal relationships that could have appeared to influence the work reported in this paper.
