# Supplementary material for: Multifaced risk factors and clinical impact of a deep Y descent in patients with heart failure irrespective of RV-PA coupling
Source: Int J Cardiol Heart Vasc. 2024 Jun 10;53:101439. doi: 10.1016/j.ijcha.2024.101439 (PMC11209010; doi:10.1016/j.ijcha.2024.101439)
Supplement: Supplementary Data 1 [file mmc1.docx]

Supplementary file

1. Jugular venous pulse waveform
2. The variance inflation factor of variables for a deep Y descent
3. The correlation matrix between selected variables for cardiac events
4. Classification of heart failure
5.
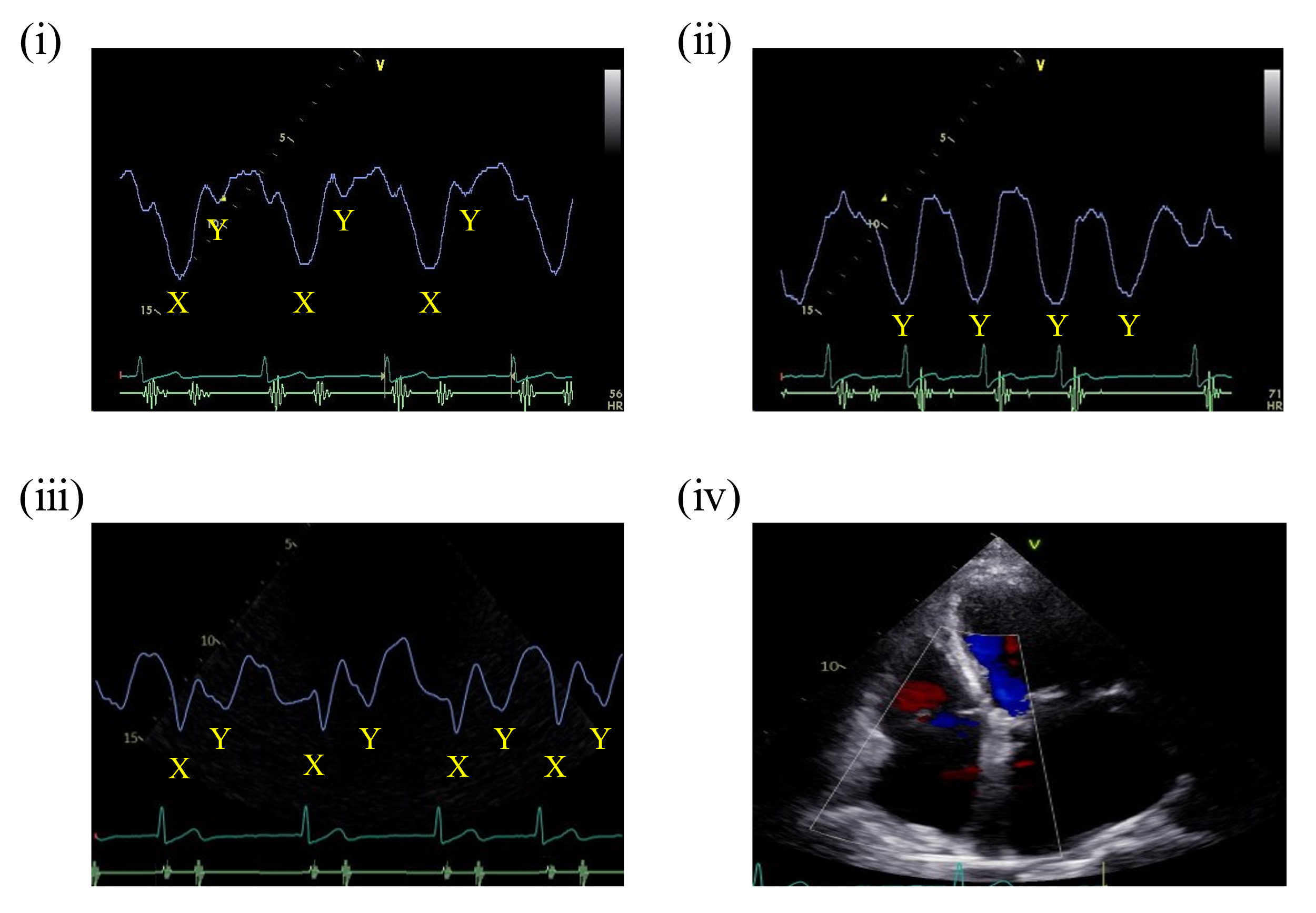
Jugular venous pulse waveform

Several typical jugular venous pulse waveforms are shown above. ⅰ) A 57-year-old woman had sinus rhythm and mild tricuspid regurgitation. The jugular venous pulse was judged as a normal Y descent. ⅱ) A 87-year-old woman had atrial fibrillation tachycardia and severe tricuspid regurgitation. The X descent was disappeared from the jugular venous pulse waveform. The patient was excluded from this study due to the inability to identify X descent and severe valvular heart disease. ⅲ) and ⅳ) A 52-year-old man had atrial fibrillation. Tricuspid regurgitation was rarely observed as shown in figure ⅳ). The waveform was judged as a normal Y descent.

1. The variance inflation factor of variables for a deep Y descent

|  | Variance inflation factor |
| --- | --- |
| Age | 1.633784 |
| Heart rate | 1.271724 |
| Hypertension | 1.178650 |
| Prior hospitalization for decompensated heart failure | 1.376298 |
| Atrial fibrillation | 2.346223 |
| ACEI/ARB | 1.266014 |
| Mineralocorticoid receptor antagonist | 1.473013 |
| Beta blockers | 1.238955 |
| Loop diuretics | 1.561524 |
| Brain natriuretic peptide | 1.353146 |
| Left atrial volume index | 1.504816 |
| Mean mitral e’ | 3.476989 |
| Mean mitral E/e’ | 2.263863 |
| Moderate mitral regurgitation | 1.336240 |
| Right ventricular outflow tract | 1.059726 |
| TAPSE/PASP ratio | 1.513695 |
| Moderate tricuspid regurgitation | 1.233731 |
| Inferior vena cava | 1.312801 |

The variance inflation factor of variables for a deep Y descent is shown in above table. The features with variance inflation factor with exceeding 10 were not observed. ACEI/ARB, angiotensin-converting enzyme inhibitors/angiotensin receptor blockers; TAPSE/PASP, tricuspid annular plane systolic excursion/ pulmonary artery systolic pressure.

1.
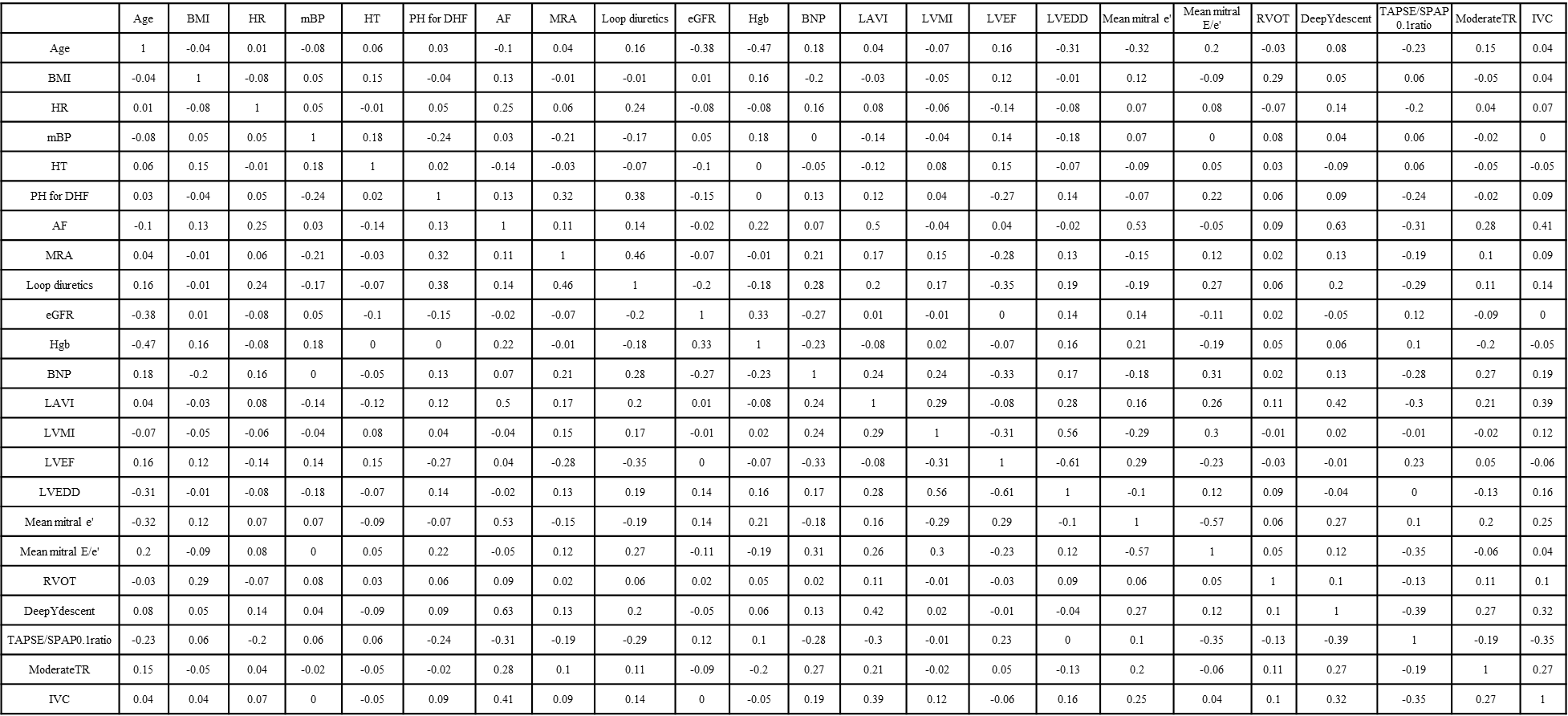
The correlation matrix between selected variables for cardiac events

The correlation matrix between selected variables for cardiac events are shown in above table. There were no factors with a strong correlation exceeding 0.7. AF, atrial fibrillation; BMI, body mass index; BNP, brain natriuretic peptide; eGFR, estimated glomerular filtration rate; Hgb, hemoglobin; HR, heart rate; HT, hypertension; IVC, inferior vena cava; LAVI, left atrial volume index; LVEDD, left ventricular end diastolic dimension; LVEF, left ventricular ejection fraction; LVMI, left ventricular mass index; mBP, mean blood pressure; MRA, mineralocorticoid receptor antagonist; PH for DHF, prior hospitalization for decompensated heart failure; RVOT, right ventricular out tract; TAPSE/SPAP, tricuspid annular plane systolic excursion; TR, tricuspid regurgitation.

4. Classification of heart failure

HFrEF

HFrEF

HFmrEF

HFpEF

HFmrEF

HFpEF

n=10

n=14

n=83

n=6

n=24

n=6

n=2

n=1

n=7

At the entry point

Prior to the entry point

Out of 350 patients, 153 had undergone echocardiography at our hospital prior to the entry point, allowing for the longitudinal observation of LVEF changes. There were 36 patients classified as heart failure with recovered ejection fraction, 107 patients classified as heart failure with unchanged ejection fraction, and 10 patients classified as heart failure with worsened ejection fraction at the entry point. HFmrEF, heart failure mid-range ejection fraction; HFpEF, heart failure with preserved ejection fraction; HFrEF, heart failure reduced ejection fraction.
